# Structural and Electrochemical Analysis of CIGS: Cr Crystalline Nanopowders and Thin Films Deposited onto ITO Substrates

**DOI:** 10.3390/nano11051093

**Published:** 2021-04-23

**Authors:** Suzan Saber, Bernabé Marí, Andreu Andrio, Jorge Escorihuela, Nagwa Khattab, Ali Eid, Amany El Nahrawy, Mohamed Abo Aly, Vicente Compañ

**Affiliations:** 1 Institut de Disseny i Fabricació, Universitat Politècnica de València, Camí de Vera s/n, 46022 Valencia, Spain; s.k.saber@hotmail.com; 2 National Research Center, 33 El Bohouth St. (Former El Tahrir St.), Dokki, Giza, Cairo 12622, Egypt; nag_khb@yahoo.com (N.K.); suzankamal85@gmail.com (A.E.); amany_physics_1980@yahoo.com (A.E.N.); 3 Departament de Física Aplicada, Universitat Jaume I, Avda. Sos Baynat s/n, 12080 Castelló, Spain; andrio@uji.es; 4 Departamento de Química Orgánica, Universitat de València, Avda. Vicent Andrés Estellés s/n, Burjassot, 46100 Valencia, Spain; 5 Chemistry Department, Faculty of Science, Ain Shams University, Cairo 11566, Egypt; aboalymoh@hotmail.com; 6 Departament de Termodinàmica Aplicada, Escola Tècnica Superior d’Enginyers Industrials (ETSII), Universitat Politècnica de València, Camí de Vera s/n, 46022 València, Spain

**Keywords:** chalcopyrite compounds, nanocrystals, hydrothermal, spin coating, EIS, conductivity

## Abstract

A new approach for the synthesis of nanopowders and thin films of CuInGaSe_2_ (CIGS) chalcopyrite material doped with different amounts of Cr is presented. The chalcopyrite material CuIn_x_Ga_1 − x_Se_2_ was doped using Cr to form a new doped chalcopyrite with the structure CuInxCr_y_Ga_1 − x − y_Se_2_, where x = 0.4 and y = 0.0, 0.1, 0.2, or 0.3. The electrical properties of CuIn_x_ Cr_y_Ga_1 − x − y_Se_2_ are highly dependent on the Cr content and results show these materials as promising dopants for the fabrication thin film solar cells. The CIGS nano-precursor powder was initially synthesized via an autoclave method, and then converted into thin films over transparent substrates. Both crystalline precursor powders and thin films deposited onto ITO substrates following a spin-coating process were subsequently characterized using XRD, SEM, HR-TEM, UV–visible and electrochemical impedance spectroscopy (EIS). EIS measurement was performed to evaluate the dc-conductivity of these novel materials as conductive films to be applied in solar cells.

## 1. Introduction

In the past decade, the photovoltaics (PV) technology has strongly evolved and even reached grid parity with other conventional energy sources [1]. In this regard, significant advances have been reported after long time of investigation in the field of thin film solar cells technology [2,3]. Although silicon has been the dominant material in the market of PV technology, others thin films options such as cadmium telluride (CdTe), copper indium gallium disulfide (CIGS2), and copper indium gallium diselenide (Cu(In,Ga)Se_2_, CIGS) materials, which are capable of maintaining constant their efficiency for more than 15 years, have recently reached conversion efficiencies of 22.1% and 22.6%, respectively [4,5]. Furthermore, other new emerging technologies such hybrid perovskite solar cells have also improved efficiencies up to 22.1% in the past years [6,7,8,9]. This massive development is mainly dependent on the economic situation, as global prices have increased in the recent years in combination with the fuel sources depletion [10].

The first practical solar cell reported in the 1950s was mainly formed of crystalline silicon and with an efficiency around 4.5% [11]. Since then, a considerable increase in the materials used in the 1970s reached tens of absorbers. Afterwards nanotechnology and materials science have grown exponentially since the 1990s. This blossoming enriched the PV field and led to significant advances in this field. The most relevant output production of that era was the synthesis of CdTe and CIGS materials [12,13,14,15]. From the point of view of material properties, the solar cells are generally divided into two basic categories: bulk and thin-film solar cells [16,17]. The first group is made of monocrystalline silicon or polycrystalline silicon materials, whereas thin film photovoltaic cells are based on solution processable semiconductors. Materials such as CIGS and other materials from the same family have emerged as promising candidates to be used in thin-film solar cells due to their high absorption coefficient, changeable bandgap, and resistance to photo-degradation [18,19,20,21]. CIGS thin films are generally fabricated using non-vacuum techniques, such as co-evaporation and sputtering techniques [22]. However, vacuum methods have several drawbacks such as high cost, difficulty to produce large area coatings and low yields. One of the critical disadvantages facing CIGS development is the multistage processes such as co-evaporation and two-step processes involving sputtering followed by a selenization stage [17,18,19,20,21,22,23,24,25,26,27,28].

On the other hand, film deposition using non-vacuum techniques are at present the most widely used in industrial applications because they offer an apparent alternative to vacuum-based processes such as colloidal methods, sol–gel [29,30], paste coating [31], inkjet printing [32], and solvothermal processes [33,34]. From all of them, the solvothermal method constitutes the most competitive process, mainly due to the low cost and high efficiency, and actually has a great demand in the production of solar cells at the industrial scale [35]. Pre-deposition, co-evaporation incorporation, and post-deposition incorporation are the main strategies of deposition generally used for thin film preparation of CIGS chalcopyrite materials [36].

The doping process reported in the literature is mostly focused on alkali doping and some for-trace metal impurities incorporated from substrate material to absorption layers [37,38]. Alternative doping agents in the CIGS layer such as Fe impurities on the photovoltaic properties of the solar cells [39,40,41], and other metals such as Mn, V, Ti, Cr, Ni, and Al have also been evaluated on the performance of the solar cells [42,43]. However, a thorough study of how these impurities can act as defects, and influence on the electronic properties of the material is still scarce for the family of CIGS compounds.

Herein, we used a pre-deposition incorporation process form the preparation of CIGS compounds, which incorporates Cr in the precursor solution to afford the starting material of precursor powders. The thermal treatment performed was provided using autoclave (solvothermal) method. This precursor powders were used in in the preparation of thin films. In particular, we focused on the preparation of CuIn_x_Cr_y_Ga_1 − x − y_Se_2_ with x = 0.4 and y = (0.0, 0.1, 0.2, 0.3) with a well-controlled particle size in the order of nanometers. The crystalline precursor powders and thin films deposited onto indium tin oxide (ITO) glass substrates using a spin-coating process were subsequently characterized using X-ray diffraction (XRD), field emission scanning electron microscopy (FE-SEM), high-resolution transmission microscopy (HR-TEM), energy dispersive X-ray spectroscopy (EDX), UV–visible and electrochemical impedance spectroscopy (EIS). The analysis of results allowed us to quantify which is the doping percentage of Cr in the CuIn_0.4_Ga_0.6_Se_2_ structure.

## 2. Materials and Methods

### 2.1. Synthesis of CuIn_x_Cr_y_Ga_1 − x_Se_2_ Nano-Crystalline Precursor Powders

CuInxGa_1 − x_Se_2_ was synthesized by the solvothermal method. For this, powders of Cu (99.9%), Se (99.99%), GaI_3_ (99%), and InCl_3_ (99.999%) from Sigma–Aldrich were used as copper, selenium, gallium, and indium sources, respectively. Cr(ClO_4_)_3_ was used as Cr^3+^ source and ethylenediamine (99.5%, Sigma–Aldrich Química SL, Madrid, Spain) was used as solvent. All compounds were introduced in a nitrogen-filled glove box and oxygen below 1 ppm. The amount of each precursors was 2 mmol of the copper elemental powder, 1 mmol of indium, gallium metal sources, and 4 mmol of selenium powder, respectively. Chromium percentage was calculated for every batch process in order to prepare different Cr and Ga series at a fixed indium percentage to get this structure CuIn_x_Cr_y_Ga_1 − x − y_Se_2_ with x = 0.4 and y = (0.0, 0.1, 0.2, 0.3).

All precursors were weighed and stirred overnight with a suitable amount of solvent. The stirred solution was then loaded into a Teflon autoclave container and annealed in an open-air oven for 36 h at 220 °C. The resultant product was washed several times using ethanol and distilled water to remove excess by-product or impurity materials. Centrifuging and ultra-sonication processes were repeated every washing time. The centrifuging process were conducted for 10 min at 4000 rpm. Finally, the completely purified precursor powders were dried at 100 °C for 2 h, and a black nano-crystalline powder of CuIn_x_Cr_y_Ga_1 − x − y_Se_2_ was obtained.

### 2.2. Synthesis of the CuIn_x_Cr_y_Ga_1 − x_Se_2_ Thin Films

All the collected synthesized precursor powders were washed several times with distilled water and ethanol in order to remove all sub product residue chemicals. These washed powders were then dried in open-air drier at 100 °C for 2 h. After this the powders were loaded into the glove box redispersion in the same preparation solvent as and left for stirring overnight then filtered using 0.2 μm mesh filters. Completely clear dissolved filtered precursors solution of the prepared nanocrystalline powders were obtained available for further processes.

Indium tin oxide (ITO) glass substrates were cut down into small pieces of the desired surface area. Cleaning process of the glass substrate were applied by washing the substrates using soap, water, ethanol, and acetone ultrasonically, dried, and then introduced into the glove box. The dissolved nanocrystalline precursor solution was then transformed to thin films by spin coating process. Metal salts nano-crystalline precursor powder to solvent ratio were taken in consideration (1:3). Supplementary Figure S1 depicts the setup use for the synthesis of CuIn_x_Cr_y_Ga_1 − x_Se_2_ thin films. Subsequent thermal treatment using a pre-heated hot plate was applied for improving the crystal structure and reach the required film thickness.

### 2.3. Characterization of CuIn_x_Ga_1 − x_Se_2_ Nano-Crystalline Powders and Thin Films

X-ray diffraction (XRD) was used to characterize the crystalline structure and phase of the prepared powder. The measurements were recorded in *2*θ range from 20 to 65° with a Rigaku Ultima IV diffractometer in the Bragg–Bentano configuration using CuKα radiation (λ = 1.54060 Å). The particle size and morphology of the resultant CIGS precursor powder were characterized by field emission scanning electron microscopy (FESEM) of model Zeiss ULTRA 55 equipped with In-Lens and secondary electrons detectors. High-resolution transmission microscopy (HRTEM, 200 KV) JEOL Model: JEM-2100F (Tokyo, Japan) were used to study the crystalline structure and morphology of nanowire arrays. The chemical composition and the purity of the samples were characterized using energy dispersive X-ray spectroscopy (EDX). Finally, an Novocontrol broadband dielectric spectrometer (BDS) (Hundsangen, Germany) integrated by an SR 830 lock-in amplifier with an alpha dielectric interface was used for measuring the conductivity of the resultant precursor powders.

### 2.4. Electrochemical Impedance Spectroscopy (EIS) Measurements

The powders conductivity in the transversal direction were measured by impedance spectroscopy in the frequency interval of 10^−1^ < f < 10^7^ Hz applying a 0.1 V signal amplitude to ensure the linear response in a range of temperature between 20 and 200 °C. The measurements were carried out in dry and wet conditions using a Novocontrol broadband dielectric spectrometer (BDS) (Hundsangen, Germany) integrated by an SR 830 lock-in amplifier with an Alpha dielectric interface was used [44,45,46]. The powders were previously dried in a vacuum cell and their thicknesses were measured afterwards using a micrometer, taking the average of 10 measurements in different parts of the surface. Next the samples were sandwiched between two gold circular electrodes coupled to the impedance spectrometer acting as blocking electrodes. Before to start the measurement the assembly powder-electrode was annealed in the Novocontrol setup under an inert dry nitrogen atmosphere. For this, firstly a temperature cycle from 20 to 200 °C and then from 200 to 20 °C, in steps of 20 °C, was carried out. After this, in a new cycle of temperature scan, the dielectric spectra were collected in each step from 20 to 200 °C, this cycle was named dry conditions. This was performed to ensure the measurements reproducibility and to eliminate the potential interference of water retained, in particular considering the hygroscopicity of the CIGS. For the measurements in wet conditions the samples were previously dissolved in bi-distilled water in the ratio 40:60 wt % (sample:water). The obtained paste was subsequently sandwiched between two gold electrodes. During the measurements in wet conditions, the sandwiched powder between the two electrodes were kept in a BDS 1308 liquid device, coupled to the spectrometer following the experimental process following a described procedure [47,48,49,50]. During the conductivity measurements, the temperature was maintained (isothermal experiments) with a stepwise of 20 °C from 20 to 200 °C controlled by a nitrogen jet (QUATRO from Novocontrol) with a temperature error of 0.1 K during every single sweep in frequency. From the frequency dependence of complex impedance Z*(ω) = Z’(ω) + j·Z”(ω), the real part of the conductivity is given as Equation (1)

(1) $$\sigma \prime (\omega )=\frac{Z\prime (\omega )\cdot L\;}{\left[{\left(Z\prime (\omega )\right)}^{2}+{\left(Z\prime \prime (\omega )\right)}^{2}\right]\cdot S}=\frac{L}{R_{0}\cdot S}$$

where L and S are the thickness and area of the sample sandwiched between the electrodes, respectively, and R_0_ the resistance of the sample.

## 3. Results and Discussion

### 3.1. Synthesis of CuIn_x_Cr_y_Ga_1 − x − y_Se_2_ Nanocrystals

**Figure 1:**
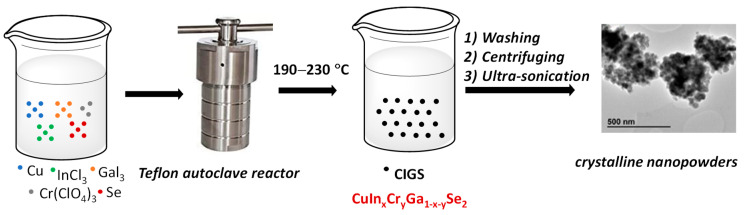
Schematic representation of the preparation of CIGS nanoparticles.

CIGS nanocrystals were synthesized via the solvothermal method by mixing the corresponding amounts of the source materials (see Materials and methods for a full description of the synthesis), in this case Cu, Se, GaI_3_, InCl_3_, and Cr(ClO_4_)_3_, at the desired stoichiometry in ethylenediamine as solvent (Figure 1). In our methodology, the In/Ga ratio was varied from 0 to 1. The solution was then loaded into a Teflon autoclave container and annealed in an open-air oven for 36 h at 220 °C. The resultant powder was washed several with ethanol and water. Then, the solid centrifugated at 4000 rpm and ultra-sonicated to finally give a black nano-crystalline powder of CuIn_x_Cr_y_Ga_1 − x − y_Se_2_.

### 3.2. Structural Study

#### 3.2.1. XRD of the Precursor Powder

**Figure 2:**
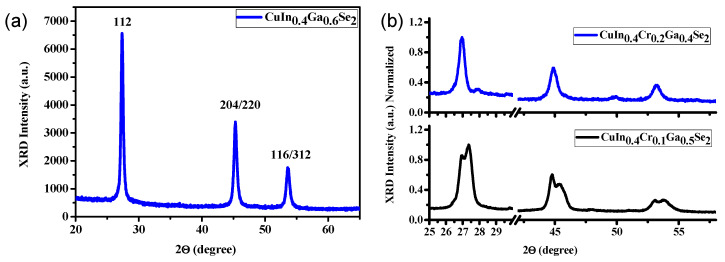
XRD patterns of (**a**) CuIn_0.4_Ga_0.6_Se_2_ precursor nanocrystals and (**b**) CuIn_0.4_Cr_0.1_Ga_0.5_Se_2_ and CuIn_0.4_Cr_0.2_Ga_0.4_Se_2_ precursor nanocrystals both prepared by autoclave hydrothermal method.

Figure 2a shows the XRD pattern of the prepared sample with a chalcopyrite structure. Three sharp diffraction peaks were obtained, which confirms a chalcopyrite (tetragonal) phase structure corresponding to CuIn_0.4_Ga_0.6_Se_2_. The diffraction peaks match well to standard CIGS file (PDF card no. 00–035–1101). The three peaks observed were identified as a pure phase of the CuIn_x_Ga_1 − x_Se_2_ chalcopyrite phase with no secondary phases. The main peaks of CIGS powders correspond to crystallographic planes labelled as (112) at 27.36°, (204)/(220) at 2θ = 45.35°, and (116)/(312) at 2θ = 53.62°, respectively, in agreement with similar materials [51].

Figure 2b displays the XRD pattern for CuIn_0.4_Cr_0.1_Ga_0.5_Se_2_ and CuIn_0.4_Cr_0.2_Ga_0.4_Se_2_ nano-powders. In the case of CuIn_0.4_Cr_0.2_Ga_0.4_Se_2_ powders, only the crystallographic peaks corresponding to chalcopyrite CIGS structure could be observed, i.e., (112) peak located at 26.94°, (204)/(220) peaks at *2*θ = 44.88°, and (116)/(312) at *2*θ = 53.22°, respectively. The main characteristic peaks for the CuIn_0.4_Cr_0.1_Ga_0.5_Se_2_ samples were located at 26.93°, 44.77°, and 53.03°, respectively, whereas they were combined with a subsidiary wurtzite Cu_2-x_Se phase located at 27.35°, 45.48°, 53.79°, respectively, shifted to higher *2*θ degrees [52]. On the other hand, the sample of CuIn_0.4_Cr_0.1_Ga_0.5_Se_2_ displayed the main characteristic peaks of chalcopyrite CIGS in addition to some wurtzite Cu_2-x_Se peaks. Herein, every peak is built up by combination of two divided peaks like camel humps (like it has been doubleted), which can be attributed to the presence of a small percent of Cr(III) ions in the crystal lattice. The incorporation of ions with three electrons in the 3d shell and spin up, and the 3d^10^ configuration of Cu(I) ion results in one unique hole in the 3d shell, because the spin of the Cu(I) ion is aligned antiparallel to that of Cr(III) ion. These holes are supposed to be delocalized and occupy the states in a narrow d band. The metallic behavior is associated with the *t_2g_* orbital of these delocalized Cu(I) holes. Therefore, we expect here a strong competition between Cu_2-x_Se wurtzite phase formation and CuIn_0.4_Cr_0.1_Ga_0.5_Se_2_, which result in the formation of both phases.

In CuIn_0.4_Cr_0.2_Ga_0.4_Se_2_ samples, where the percentage of Cr(III) is equal to 20% which is higher than in CuIn_0.4_Cr_0.1_Ga_0.5_Se_2_ samples, the increase in Cr(III) content affects to the CuIn_x_CryGa_1 − x − y_Se_2_, (x = 0.4 and y = 0.0, 0.1, 0.2, 0.3) chalcopyrite phase formation. The CuIn_0.4_Cr_0.3_Ga_0.3_Se_2_ has 30% of Cr(III) ions in addition to Ga(III) ions, which leads to a competition between Cr(III) and Ga(III) ions rather than the stated competition between Cr(III) and Cu(I) ions.

The mean crystallite size of the prepared polycrystalline CIGS was calculated according to Scherer’s formula (Equation (2))

(2) $$D=\frac{K\lambda }{\beta cos\theta }$$

where *β* is the full width at half maximum (FWHM), λ is the wavelength of X-rays (1.5418 Å), K is the Scherer’s constant which depends on the crystallite shape and is close to 1, and θ is the Bragg angle at the center of the peak.

The obtained XRD patterns can be assigned to a tetragonal CuIn_0.4_Ga_0.6_Se_2_ crystallographic phase (PDF card no. 00–035-1101) with a preferred orientation along the (112) plane. No other stoichiometric composition of CIGS was obtained. The well-defined and sharp diffraction peaks indicated that the material showed good crystallinity and evidences the absence of any additional diffraction peaks of possible binary phases or impurities. Table 1 shows the measured position of the 112-diffraction peak, its FWHM, and the crystalline size for all CuInGaSe_2_ with different contents of Cr. The crystalline size ranges from 10 to 20 nm depending on the Cr content in the powders.

#### 3.2.2. XRD of CIGS Thin Films

**Figure 3:**
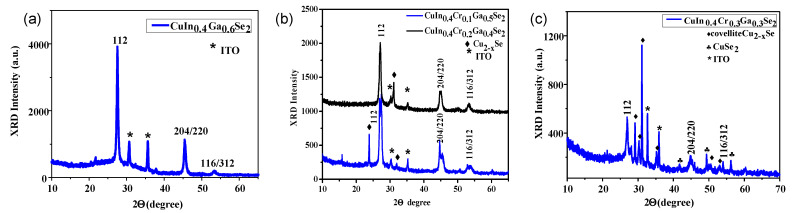
XRD patterns of thin film deposited onto ITO by spin coating process of (**a**) CuIn_0.4_Ga_0.6_Se_2_ substrate, (**b**) CuIn_0.4_Cr_0.1_Ga_0.5_Se_2_ and CuIn_0.4_Cr_0.2_Ga_0.4_Se_2_, (**c**) CuIn_0.4_Cr_0.3_Ga_0.3_Se_2_.

After characterizing the CIGS nanoparticles, we evaluated their structure after deposition on indium tin oxide (ITO) glass substrates and fabrication of thin films by spin coating process. Figure 3a shows the XRD graph of the thin film CuIn_0.4_Ga_0.6_Se_2_ on the ITO substrate. XRD patterns are consistent with chalcopyrite (tetragonal) crystal structure and exhibit a peak broadening due to their nanoscale crystal size. The diffraction peaks shift to lower *2*θ degrees with decreasing Ga content by incorporation of Cr(III) ions in Ga(III) sites. The increase in the lattice spacing is due to smaller Cr atoms substituting for larger Ga atoms which in turn lead to a significant change in lattice parameters values.

The stoichiometric compound Cu(In,Ga)Se_2_ crystallizes in the tetragonal chalcopyrite type crystal structure with space group *I*-42*d* (122). Within this crystal structure the monovalent cations of Cu(I) occupy the 4a site (0 0 0), the trivalent cations of In(III) and Ga(III) are located on the 4b position (0 0 ½) and the selenium anions are on the 8d site (x ¼ ⅛). The cations are tetrahedrally coordinated by the anions and vice versa, substitution of Ga(III) ions with Cr affect lattice values due to the difference in atomic radius of both Cr and Ga atoms. In general, the absorbing layers of Cu(In,Ga)Se_2_ exhibit a poor copper composition (Cu/(In + Ga) < 1), whereby the chalcopyrite-like crystalline structure persists together with the occupation of the sites of Wyckoff. This can cause specific changes due to possible defects in the material. As a consequence, there is a strong correlation between the concentration of these defects and the electronic and optical properties of the material, which can be especially interesting to adapt to high-efficiency photovoltaic devices. All the doped samples with Cr showed an enhancement in the lattice value corresponding to the FWHM value for CuIn_0.4_Ga_0.4_Se_2_. We notice the main characteristic peaks of CuIn_0.4_Ga_0.6_Se_2_ and those characteristics of ITO, which were located at *2*θ = 30.58° and 35.48°, respectively [53]. A well-defined crystallographic chalcopyrite structure with all preferred orientation a long 112, (204)/(220) and (116)/(312) for CuIn_0.4_Ga_0.4_Se_2_ at *2*θ = 27.49°, *2*θ = 45.37°, and *2*θ = 53.36° is clearly observed.

The XRD patterns of the thin film for CuIn_0.4_Cr_0.1_Ga_0.5_Se_2_ and CuIn_0.4_Cr_0.2_Ga_0.4_Se_2_ on ITO substrate are displayed in Figure 3b. We notice those main characteristic peaks of CuIn_0.4_Cr_0.2_Ga_0.4_Se_2_ clearly at *2*θ = 27.18°, *2*θ = 44.68°, and *2*θ = 53.33° and those characteristics of ITO. Wurtzite Cu2-xSe phase was observed by thin film formation in both of CuIn_0.4_Cr_0.1_Ga_0.5_Se_2_ and CuIn_0.4_Cr_0.2_Ga_0.4_Se_2_, whereas it is observed in a small percent for CuIn_0.4_Cr_0.2_Ga_0.4_Se_2_ thin film samples. The CuIn_0.4_Cr_0.1_Ga_0.5_Se_2_ thin film structure characteristic peaks were observed at 2θ = 26.72° and 27.36°, 2θ = 44.52° and 45.45°, and 2θ = 52.99° and 53.67° for 112, (204)/(220) and (116)/(312), respectively. The peaks duplicity is clearly observed for CuIn_0.4_Cr_0.1_Ga_0.5_Se_2_ thin film structure than those of CuIn_0.4_Cr_0.2_Ga_0.4_Se_2_. On the other hand the pure well defined highly oriented 112, (204)/(220) and (116)/(312) peaks for CuIn_0.4_Cr_0.2_Ga_0.4_Se_2_ were identified. As shown in Figure 3c, which shows the XRD graph of the thin film CuIn_0.4_Cr_0.3_Ga_0.3_Se_2_ on ITO substrate, the characteristic peaks of CuIn_0.4_Cr_0.3_Ga_0.3_Se_2_ at *2*θ = 26.96°, *2*θ = 44.78°, and *2*θ = 53.94° were observed. Mixture of copper selenide phases are very apparent in the XRD pattern for the CuIn_0.4_Cr_0.3_Ga_0.3_Se_2_ sample—i.e., covellite Cu_2-x_Se and CuSe_2_—which is due to the stated confliction between both Cr(III) and Cu(I) ions at definite amount of incorporated Cr(III) ions that results in impurity phase formation structure. Using Scherrer formula the crystal size of all the prepared thin films of CuIn_0.4_Ga_0.4_Se_2_, CuIn_0.4_Cr_0.1_Ga_0.5_Se_2_, CuIn_0.4_Cr_0.2_Ga_0.4_Se_2_, and CuIn_0.4_Cr_0.3_Ga_0.3_Se_2_ were calculated. It was found that the crystal size is equal to 20, 10, 17, and 18 nm for all the prepared samples of the structure where x = 0.4 and y = 0.0, 0.1, 0.2, 0.3, respectively. As inferred from Table 2, the FWHM of CuIn_0.4_Cr_0.1_Ga_0.5_Se_2_ thin film sample show remarkable higher value than those of CuIn_0.4_Ga_0.6_Se_2_, CuIn_0.4_Cr_0.2_Ga_0.4_Se_2_, and CuIn_0.4_Cr_0.3_Ga_0.3_Se_2_. This remarkable increase of FWHM value is similar to those FWHM values of the grown samples, which confirms the structure defects and the stablished competition between Cr(III) and Cu(I) ions in the unit cell structure of those with low Cr% = 10, and Cr% = 30. The weak broad peak in the XRD pattern of this CIGS nanoparticle film is indexed to the (112) plane of a CIGS chalcopyrite crystal structure, which also implies that the film is very thin and not high in crystallinity due to its low annealing temperature [54].

### 3.3. FE-SEM Analysis

#### 3.3.1. FE-SEM Analysis of CIGS Nano-Crystalline Powders

**Figure 4:**
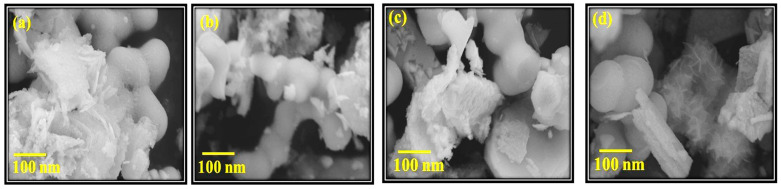
FE-SEM images for (**a**) CuIn_0.4_Ga_0.6_Se_2_, (**b**) CuIn_0.4_Cr_0.1_Ga_0.5_Se_2_, (**c**) CuIn_0.4_Cr_0.2_Ga_0.4_Se_2_, and (**d**) CuIn_0.4_Cr_0.3_Ga_0.3_Se_2_ nano-crystalline powders at 100 nm.

The particle size and morphology of the resultant CIGS was evaluated by FE-SEM. Figure 4 shows FE-SEM images for CuIn_x_Cr_y_Ga_1 − x − y_Se_2_ where x = 0.4 and y = (0.0, 0.1, 0.2, 0.3) nanocrystals (see Supplementary Figure S2 for images at 2 μm). According to FE-SEM images of the nano-crystalline precursor powders, CuIn_0.4_Ga_0.6_Se_2_, CuIn_0.4_Cr_0.1_Ga_0.5_Se_2_, CuIn_0.4_Cr_0.2_Ga_0.4_Se_2_, and CuIn_0.4_Cr_0.3_Ga_0.3_Se_2_ contain agglomerates of spherical particles and some tetragonal and plate like shapes. Some tetrahedron hexagonal phases were determined in both CuIn_0.4_Cr_0.1_Ga_0.5_Se_2_, CuIn_0.4_Cr_0.2_Ga_0.4_Se_2_, and CuIn_0.4_Cr_0.3_Ga_0.3_Se_2_, in consistency with the XRD results. Those tetrahedron and hexagonal structures refer to the presence of Cu_2-x_Se and CuSe_2_ phases in a complete matching with the XRD peaks referred to CuSe phases. Those tetrahedron and hexagonal structures refer to the presence of Cu_2-x_Se and CuSe_2_ phases in a complete matching with the XRD peaks referred to CuSe phases [55]. For the FE-SEM images of CuIn_0.4_Ga_0.6_Se_2_ nano-crystalline powders only those spherical of the chalcopyrite structure are present. No other morphologies have been observed, which in turn confirms the XRD data and of crystal structure and particle size calculations.

The structures of the prepared precursor powders provide a promising feedback about the expected thin films surface morphology, which resemble the nano crystalline precursor powders. The average particle size is approximately (10–20 nm) in accordance with the HRTEM and XRD crystallite size. Such characteristic properties will play a vital role in the deposition process of the CIGS thin films. Particle size, morphology, uniformity, homogeneity, adhesion and cohesion of the prepared precursor powders; all these properties will affect the nature of the resultant films by any formation process. In general, one aims for particulate precursor powders with a few hundred nanometers and good uniformity in particles shape will lead to a sufficient adhesion to the substrate and sufficient cohesion of one particle to another, as shown in Figure 4.

#### 3.3.2. FE-SEM Analysis of CIGS Thin Films

**Figure 5:**
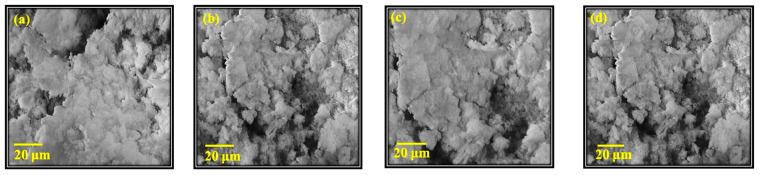
FE-SEM images for (**a**) CuIn_0.4_Ga_0.6_Se_2_, (**b**) CuIn_0.4_Cr_0.1_Ga_0.5_Se_2_, (**c**) CuIn_0.4_Cr_0.2_Ga_0.4_Se_2_, and (**d**) CuIn_0.4_Cr_0.3_Ga_0.3_Se_2_ thin films at 20 μm.

FE-SEM images for the prepared thin films containing CuIn_0.4_Ga_0.6_Se_2_, CuIn_0.4_Cr_0.1_Ga_0.5_Se_2_, CuIn_0.4_Cr_0.2_Ga_0.4_Se_2_, and CuIn_0.4_Cr_0.3_Ga_0.3_Se_2_ at 20 μm are shown in Figure 5. All films displayed a homogenous nano-crystalline particle distribution after deposition using the spin-coating process.

### 3.4. HR-TEM Analysis

**Figure 6:**
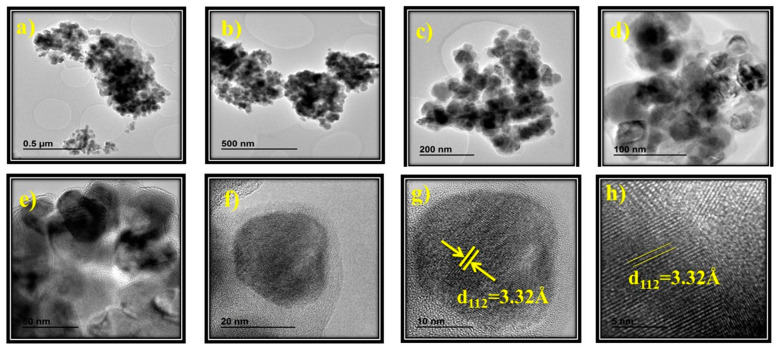
(**a**–**f**) HR-TEM images for CuIn_0.4_Ga_0.6_Se_2_ nanocrystals and (**g**,**h**) d-spacings of chalcopyrite (tetragonal) CuIn_0.4_Ga_0.6_Se_2_.

Figure 6 shows HR-TEM images of the CuIn_0.4_Ga_0.6_Se_2_ at different magnification scales. The d-spacing between crystallographic planes is shown in Figure 6g,h, and corresponds to 3.32 Å. This lattice spacing corresponds to tetragonal CuIn_x_Ga_1 − x_Se_2_ in accordance with the XRD peaks of the chalcopyrite CuIn_x_Ga_1 − x_Se_2_ [56].

Therefore, we can conclude that the crystallinity of the tetragonal chalcopyrite CuIn_0.4_Ga_0.6_Se_2_ maintains a structure basically composed by nanocrystals of approximately less than 20 nm in diameter. The d-spacings observed in HR-TEM images are also consistent with tetragonal CuIn_x_Ga_1 − x_Se_2_ chalcopyrite phase and no other crystal phases were observed in accordance with the XRD pattern of the prepared powder. The HR-TEM images show well defined small grains of a few tens of nanometers in agreement with the observed results by FE-SEM (Figure 5). The measured lattice spacing of 3.32 Å, observed in the highest magnification HR-TEM images (Figure 6g,h), matches well with the interplanar distance between the (112) crystallographic planes.

### 3.5. EDX Analysis

Energy dispersive X-ray spectroscopy (EDX) analysis of the CIGS nanocrystals was performed, and the average atomic ratios are shown in Table 3. EDX results reveal that the prepared nano-crystalline powders contain certain amount of the constituent elements in accordance with the desired structures (Supplementary Figure S3). The measured stoichiometric atomic ratios 0.87:0.4:0.6:1.98 for copper, indium, gallium, and selenium obtained by EDX are closer to the expected. However, a slight decrease of Cu and Se was found in the resultant composition in addition to an observed decrease in Cu content. The slightly copper-poor composition is beneficial for the formation of *p*-type semiconductor. The proposed composition of the prepared nanocrystal precursor powder in the first sample has a molar Cu/In/Ga/Se ratio of 1.0:0.4:0.6:2.0 with a fixed In ratio for all prepared series with incorporation of Cr atoms in Ga sites.

We observed a copper deficiency from 1.0 to 0.87, which indicates that the prepared CuIn_0.4_Ga_0.6_Se_2_ are *p*-type materials. A comparison between the proposed precursor composition and the measured EDX composition is indicated in Table 3. Although the control of the composition ratios in this type of chalcopyrites is difficult, we have been able, with this hydrothermal autoclave method, to optimize the resultant product ratios of In and Ga by controlling several factors such as metal source, time of stirring and preparation temperature. For CuIn_0.4_Cr_0.1_Ga_0.5_Se_2_ sample the proposed composition of the prepared precursor (atomic ratio of Cu/In/Cr/Ga/Se) is equal to 1:0.4:0.1:0.5:2 with an atomic percentage equal 25:10:2.5:12.5:50. The resultant atomic ratio was equal to 1.3:0.4:0.07:0.53:2.3 with an atomic ratio equal 29.44:8.8:1.58:11.49:48.69 for Cu, In, Cr, Ga, and Se, respectively. A slight increase in both Cu and Se atomic ratios is observed which in accordance with the XRD results for the presence of wurtzite Cu_2-x_Se phase in competition with the CuIn_0.4_Cr_0.1_Ga_0.5_Se_2_ chalcopyrite phase formation. This can be supposed also to the formation of CuIn_0.4_Cr_0.1_Ga_0.5_Se_2_ quartzite phase also noticed, but in total Ga + Cr = Ga main atomic ratio before substitution and In ratio is completely fixed which in turn does not affect the chalcopyrite phase formation even in the formation of the subsidiary phase as we explained in XRD part.

For CuIn_0.4_Cr_0.2_Ga_0.4_Se_2_ we noticed that the resulted composition atomic ratios for Cu, In, Cr, Ga, and Se were equal to 26.9:9.9:3.2:9.9:50.1 with an atomic ratio equal to (1.2:0.43:0.14:0.43:2.3). The proposed composition for this structure were (1:0.4:0.2:0.4:2) with an atomic ratio equal to (25:10:5:10:50). It is noticed that an equality in both In and Ga atomic ratios, slight decrease in Cr content than proposed it should work as 20% atomic ratio and it was found equal to 14%. This decrease in Cr content does not affects the chalcopyrite crystal structure, in contrast, it gives a stability with preferred orientation to the structure.

Mixture of different copper selenide phases appear as secondary phases with the determined chalcopyrite tetragonal structure of CuIn_0.4_Cr_0.3_Ga_0.3_Se_2_. The proposed composition for this structure were (1:0.4:0.3:0.3:2) with an atomic ratio equal to (25:10:7.5:7.5:50). Herein, equal amounts of Ga and Cr atoms were incorporated together in the studied phase with a noticeable variation in both Cu and Se atomic ratios, respectively. We found that the composition for Cu, In, Cr, Ga, and Se was equal to 33.60:7.22:4.90:6.09:48.19 with an atomic ratio of (1.8:0.4:0.27:0.33:2.6).

### 3.6. Optical Properties

Optical properties have been investigated from the transmittance spectra. All the CuIn_x_Cr_y_Ga_1 − x − y_Se_2_ thin films exhibited broad absorption in the visible region. The absorption coefficients were obtained for thin films samples prepared using spin-coating process with nanocrystals.

Near the absorption edge or in the strong absorption zone of the transmittance spectra of materials, the absorption coefficient is related to the optical energy gap, *E_g_*, which can be determined by the Tauc’s equation [57], Equation (3)

(3) $$\alpha =\frac{A{\left(h\nu -E_{g}\right)}^{n}}{h\nu }$$

where A is a constant, h is the Planck constant, ν is frequency, and *n* is an index that characterizes the optical absorption process and is equal to 2 for direct allowed transitions and 0.5 for indirect allowed transitions.

**Figure 7:**
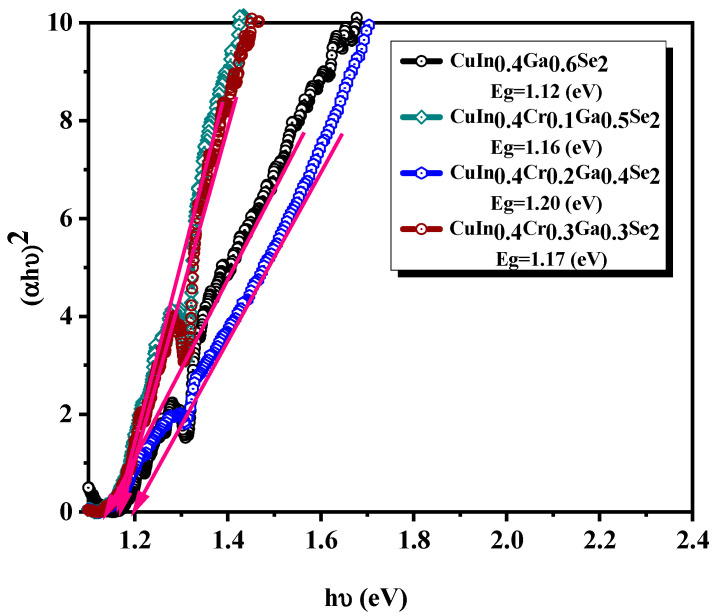
Tauc’s plot for CuIn_0.4_Ga_0.6_Se_2_, CuIn_0.4_Cr_0.1_Ga_0.5_Se_2_, CuIn_0.4_Cr_0.2_Ga_0.4_Se_2_, and CuIn_0.4_Cr_0.3_Ga_0.3_Se_2_ precursor powders.

The Tauc’s plot for CuIn_x_Cr_y_Ga_1 − x − y_Se_2_ thin films are displayed in Figure 7. The shape of Tauc’s plot indicates that the deposited CuIn_x_Cr_y_Ga_1 − x − y_Se_2_ thin films possessed a direct band gap. Extrapolation of the straight line to zero absorption coefficient (*α* = 0) allows an estimation of *E_g_*. For that, the band gaps were obtained by plotting (αhυ)^2^ vs. the energy in eV and extrapolating the linear part of the spectrum (hυ).

The prepared CuIn_0.4_Ga_0.6_Se_2_, CuIn_0.4_Cr_0.1_Ga_0.5_Se_2_, CuIn_0.4_Cr_0.2_Ga_0.4_Se_2_, and CuIn_0.4_Cr_0.3_Ga_0.3_Se_2_ thin films with *E_g_* of 1.12, 1.16, 1.20, and 1.17 eV, respectively, which are close to the optimum value for solar photoelectric conversion of 1.5 eV. The differences of the band gaps and absorption spectra of the thin films was caused by the changing particle size and morphology of the prepared CuIn_0.4_Ga_0.6_Se_2_, CuIn_0.4_Cr_0.1_Ga_0.5_Se_2_, CuIn_0.4_Cr_0.2_Ga_0.4_Se_2_, and CuIn_0.4_Cr_0.3_Ga_0.3_Se_2_ thin films with composition difference. Although composition dependency of *E_g_* has been observed for other semiconductor particles like CZTS and CuInS_2_, there has been scarce investigation of the influence of the particle composition on light−electricity conversion efficiency, especially for those new prepared CuIn_0.4_Ga_0.6_Se_2_, CuIn_0.4_Cr_0.1_Ga_0.5_Se_2_, CuIn_0.4_Cr_0.2_Ga_0.4_Se_2_, and CuIn_0.4_Cr_0.3_Ga_0.3_Se_2_ thin films.

### 3.7. Dielectric Spectra Analysis

**Figure 8:**
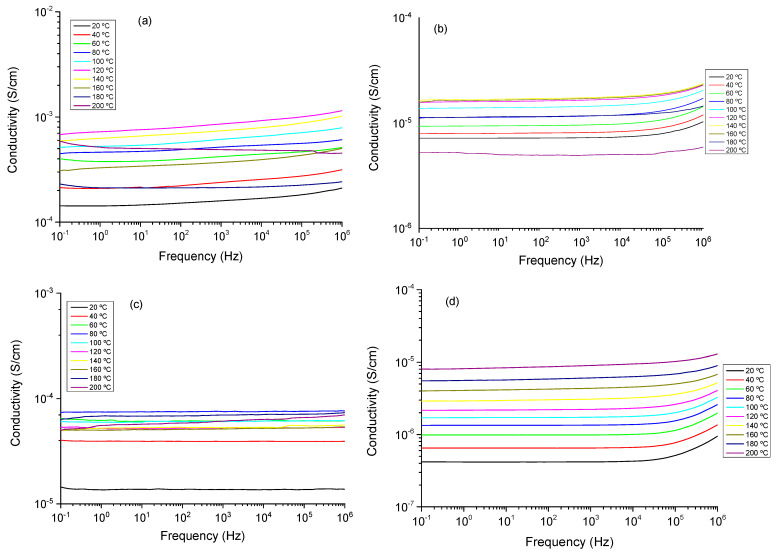
Double logarithmic plot of the real part of the conductivity versus frequency for (**a**) CuIn_0.4_Ga_0.6_Se_2_, (**b**) CuIn_0.4_Cr_0.1_Ga_0.5_Se_2_, (**c**) CuIn_0.4_Cr_0.2_Ga_0.4_Se_2_, and (**d**) CuIn_0.4_Cr_0.3_Ga_0.3_Se_2_ precursor powders obtained in dry conditions.

**Figure 9:**
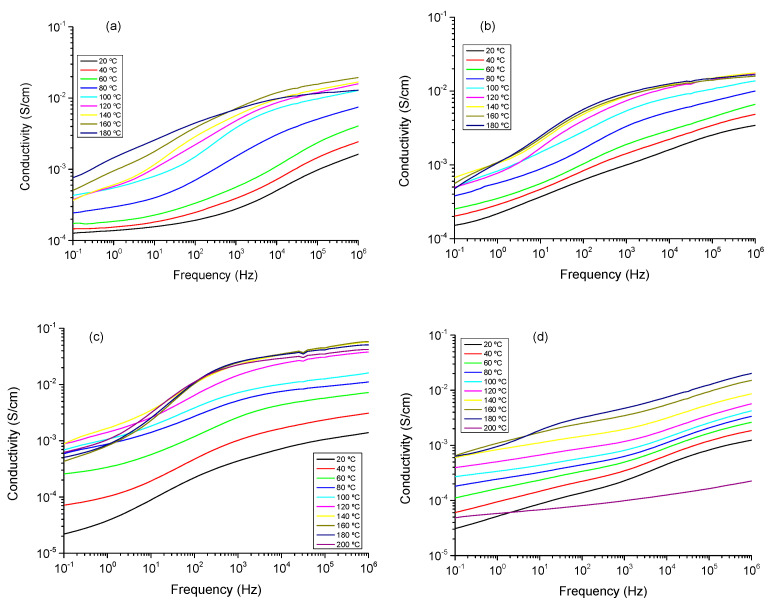
Double logarithmic plot of the real part of the conductivity versus frequency for (**a**) CuIn_0.4_Ga_0.6_Se_2_, (**b**) CuIn_0.4_Cr_0.1_Ga_0.5_Se_2_, (**c**) CuIn_0.4_Cr_0.2_Ga_0.4_Se_2_, and (**d**) CuIn_0.4_Cr_0.3_Ga_0.3_Se_2_ precursor powders obtained in wet conditions.

In order to get information about the behavior of the ionic conductivity, electrochemical impedance spectroscopy measurements were performed on the powdered solid compounds, namely, CuIn_0.4_Cr_0.1_Ga_0.5_Se_2_, CuIn_0.4_Cr_0.2_Ga_0.4_Se_2_, and CuIn_0.4_Cr_0.3_Ga_0.3_Se_2_. The measurements were carried out at different temperatures (from 20 to 200 °C) to obtain information on the samples’ conductivity. The study of the conductivity was analyzed in terms of the corresponding Bode diagrams, which are shown in Figure 8 (dry conditions) and Figure 9 (wet conditions), respectively. These plots display the variation of the conductivity with the frequency, by. plotting the double logarithmic plot of the conductivity in S/cm versus frequency in (Hz) of each sample in all range of temperatures.

In the measurements in dry conditions (Figure 8), the conductivity σ’ is characterized in the Bode plot by a plateau, where the phase angle tends to zero, the imaginary part of the impedance will be zero, and then the corresponding conductivity represents the direct-current conductivity (σdc) of the sample. A close inspection at Figure 8 revealed that samples CuIn_0.4_Cr_0.1_Ga_0.5_Se_2_, CuIn_0.4_Cr_0.2_Ga_0.4_Se_2_, and CuIn_0.4_Cr_0.3_Ga_0.3_Se_2_ possess a conductivity, practically constant in all the range of frequencies for all temperatures under study, which is a typical behavior for a conductor. Similar results have been observed in samples of multilayer graphene in polypropylene nanocomposites [58]. This can be explained as a Debye relaxation due to the motion and reorientation of the dipoles and localized charges as consequence of the electric field applied, which dominates the dc-conductivity [59,60]. For all temperatures under study, conductivity increases with the temperature following the trend, σ’ (CuIn_0.4_Ga_0.6_Se_2_) > σ’ (CuIn_0.4_Cr_0.2_Ga_0.4_Se_2_) > σ’ (CuIn_0.4_Cr_0.1_Ga_0.5_Se_2_) > σ’ (CuIn_0.4_Cr_0.3_Ga_0.3_Se_2_).

As shown in measurements in wet conditions (Figure 9), a deviation from σdc in the spectrum of the conductivity can be seen at low frequency values. This effect is attributed to the electrode polarization (EP) effect because of the blocking electrodes, and produced by mobile charge accumulation. However, it is only observed in the measurements realized below wet conditions [61,62]. The conductivity also exhibits a phenomenon of dispersion that obeys a behavior described by Jonscher law [63,64], given by: σ(ω) = σdc +σac, being σdc the dc conductivity and the alternating-current σdc = Aω*^m^*, where A is a factor dependent of the temperature and *m* for an ideal Debye dielectric and ideal ionic type crystals are 1 and 0, respectively.

**Figure 10:**
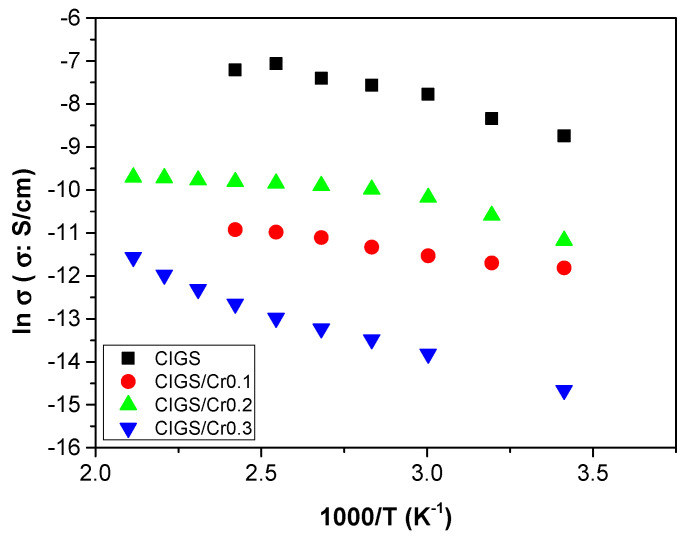
Temperature dependence of conductivity obtained from Bode diagram for all the samples studied.

Experimental data of the conductivity were fitted to the Jonscher law and the parameters are shown in Table 4. As we can see in Figure 10, only two different regions are present. On the one hand, at moderate and low frequencies where the dc-conductivity will be determined (Cond), and the region of sub-diffusive conductivity (SD), observed in the interval between 10^5^ and 10^7^ Hz for the samples CuIn_0.4_Ga_0.6_Se_2_, CuCr_0.1_In_0.4_Ga_0.5_Se_2_, and CuCr_0.3_In_0.4_Ga_0.3_Se_2_ respectively. Is important highlight that the sample, CuCr_0.2_In_0.4_Ga_0.4_Se_2_, displays a behavior of a pure conductor in all the range of frequencies under all temperatures under study [65,66,67].

In our samples, the values fitted for the parameter *m*, takes values between 0.4 and 1 for all the samples except in case of sample CuCr_0.2_In_0.4_Ga_0.4_Se_2_, where the value was 0, corresponding to an ideal conductor type in all the range of temperatures. For the other samples, the values obtained from the fit in the high frequency region which can be due to the reorientation motion of dipoles and more likely to the motion of the localized charges, which in the beginning are dominates over the dc-conductivity [65,66,67]. From Figure 9, we can see that the conductivity is a function of the amount of fillers that we have incorporated in the powdered. On the other hand, for CuCr_0.2_In_0.4_Ga_0.4_Se_2_, nanocomposite we observe that conductivity is practically constant in all the range of frequencies, only at frequencies higher than 10^6^ Hz and some temperatures, the behavior of the sample shown a cut-off frequency where it starts increasing with the frequency. For the other samples, we can see that the real part of the conductivity is constant at the low frequencies region until a cut-off frequency where it starts increasing with the frequency, as if the sample were a capacitor. The value of σ constant means that the CIGS has only a resistive contribution and its value represents the electrical conductivity of the sample. In Table 4, the values of the conductivity for the samples and the Jonscher parameters are shown.

As displayed in Figure 9, which shows the conductivity in wet conditions, it can be observed the temperature variations of the conductivity. In this plot, we can see at low temperatures σ_dc_ depends notably on the frequency and this effect tends to disappear when the temperature increases, but in our samples this behavior is not well developed due to the non-reproducibility of the measurements in wet conditions. As expected, the conductivity of all samples is strongly humidity dependent [65], increasing around two orders of magnitude, depending on the sample at each temperature. For example, for the sample CuCr_0.2_In_0.4_Ga_0.4_Se_2_ the conductivity at 60 °C is around 6.2 × 10^−5^ S/cm, however in wet conditions at the same temperature the conductivity was 5.7×10^−3^ S/cm. In all the range of temperatures studied the conductivity increase with the temperature following the trend: σ’ (CuIn_0.4_Ga_0.6_Se_2_) > σ’ (CuIn_0.4_Cr_0.2_Ga_0.4_Se_2_) > σ’ (CuIn_0.4_Cr_0.1_Ga_0.5_Se_2_) > σ’ (CuIn_0.4_Cr_0.3_Ga_0.3_Se_2_).gure 10 shows the dependence of the conductivity measured in wet conditions with temperature. From this figure, we can see that conductivity follows a typical Arrhenius behavior with two different behaviors: one where the conductivity increases with increasing temperature, between 20 and 120 °C, following the Equation (4)

(4) $$\ln\sigma_{dc}=\ln\sigma_{\infty }-\frac{E_{act}}{RT}$$

and other behavior for temperatures above 120 °C, where the conductivity begins to decrease due possibly to the dehydration of the sample.

From the slopes of the fits obtained following Equation (4), we calculated the activation energy (*E_act_*) associated to the conduction process. The values obtained followed the trend *E_act_*(CuCr_0.2_In_0.4_Ga_0.4_Se_2_) = (5.5 ± 0.2) kJ/mol < *E_act_*(CuCr_0.1_In_0.4_Ga_0.4_Se_2_) = (7.60 ± 0.15) kJ/mol < *E_act_*(CuCr_0.3_In_0.4_Ga_0.4_Se_2_) =(18.8 ± 0.2) kJ/mol < *E_act_*(CuIn_0.4_Ga_0.4_Se_2_) = (24.8 ± 0.4) kJ/mol. These results indicate that all the CIGS doped by chromium have low activation energy than CuIn_0.4_Ga_0.4_Se_2_ thin films being the best doping percentage of Cr equal to 20% the optimum.

## 4. Conclusions

In conclusion, fabrication of low-cost chalcopyrite CIGS semiconductor material using Cr as a dopant yielded an excellent well-arranged crystalline structure with the same properties and small quantities of the high-cost Ga content. Incorporation of the Cr metal, as a doping agent, in the preparation solution of the metallic salt mixture stands as a pre-deposition process type of incorporation. Resulted precursor powders in nano-sized range need to be treated in a good manner which ensures formation of excellent adhesive solution. Spin coating process was applied to get the desired new doped Cr:CIGS chalcopyrite thin films. Electrochemical impedance spectroscopy measurements confirmed that those prepared doped Cr:CIGS with different percentages are promising semiconductor materials, as inferred from the experimental data which showed that the conductivity values increase with the temperature following the trend σ’ (CuIn_0.4_Ga_0.6_Se_2_) > σ’ (CuIn_0.4_Cr_0.2_Ga_0.4_Se_2_) > σ’ (CuIn_0.4_Cr_0.1_Ga_0.5_Se_2_) > σ’ (CuIn_0.4_Cr_0.3_Ga_0.3_Se_2_). The activation energy of the powder CuCr_0.2_In_0.4_Ga_0.4_Se_2_ was 5.5 ± 0.2 kJ/mol and consequently more stable in all the range of temperatures; therefore, the optimal doping percentage of Cr was determined to be equal at 20%. Therefore, CuIn_0.4_Cr_0.2_Ga_0.4_Se_2_ thin film can be an excellent material for solar cell applications.

## Figures and Tables

**Table 1:** XRD parameters and crystallite size of CIGS nano-crystalline precursor powders

Sample Name	hkl	*2*θ (°)	FWHM	Crystallite Size (nm)
CuIn_0.4_Ga_0.6_Se_2_	112	27.36	0.39	20.3 ± 0.6
CuIn_0.4_Cr_0.1_Ga_0.5_Se_2_	112	26.92	0.83	10.2 ± 1.2
CuIn_0.4_Cr_0.2_Ga_0.4_Se_2_	112	26.94	0.48	17.4 ± 0.8
CuIn_0.4_Cr_0.3_Ga_0.3_Se_2_	112	27.59	0.41	18.1 ± 0.5

**Table 2:** XRD parameters and crystallite size of CIGS nano-crystalline thin films for the 112 peak

Sample Name	*2*θ (°)	FWHM	Crystallite Size (nm)
CuIn_0.4_Ga_0.6_Se_2_	27.5	0.40	20.2 ± 1.3
CuIn_0.4_Cr_0.1_Ga_0.5_Se_2_	27.0	0.80	10.2 ± 0.5
CuIn_0.4_Cr_0.2_Ga_0.4_Se_2_	27.2	0.50	17.1 ± 1.2
CuIn_0.4_Cr_0.3_Ga_0.3_Se_2_	26.7	0.44	18.0 ± 1.1

**Table 3:** Composition of nano-crystalline precursor powders according to EDX analysis

Sample	Cu (%)	In (%)	Cr (%)	Ga (%)	Se (%)
CuIn_0.4_Ga_0.6_Se_2_	22.6	9.7	-	16.3	51.4
CuIn_0.4_Cr_0.1_Ga_0.5_Se_2_	29.4	8.8	1.6	11.5	48.7
CuIn_0.4_Cr_0.2_Ga_0.4_Se_2_	26.9	9.9	3.2	9.9	50.1
CuIn_0.4_Cr_0.3_Ga_0.3_Se_2_	33.6	7.2	4.9	6.1	48.2

**Table 4:** Jonscher parameters obtained by fitting the equation σ(ω) = σ0 + Aω*^m^* to conductivity data, for some indicated temperature in all studied samples

Cr = 0.0	σ_dc_ [S cm^−1^]	A	*m*	Cr = 0.1	σdc [S cm^−1^]	A	*m*
T = 20 °C	1.6 × 10^−4^	10^−6.96^	0.39	T = 20 °C	7.4 × 10^−6^	10^−10.5^	0.73
T = 60 °C	4.2 × 10^−4^	10^−6.20^	0.32	T = 60 °C	9.8 × 10^−6^	10^−9.98^	0.68
T = 100 °C	6.1 × 10^−4^	10^−5.68^	0.29	T = 100 °C	1.5 × 10^−5^	10^−9.07^	0.57
T = 160 °C	3.7 × 10^−4^	10^−6.17^	0.34	T = 160 °C	1.8 × 10^−5^	10^−9.70^	0.66
**Cr = 0.2**	**σdc [S cm^−1^]**	**A**	***m***	**Cr = 0.3**	**σdc [S cm^−1^]**	**A**	***m***
T = 20 °C	1.4 × 10^−5^	0	0	T = 20 °C	4.3 × 10^−7^	10^−11.59^	0.77
T = 60 °C	6.2 × 10^−5^	0	0	T = 60 °C	1.0 × 10^−6^	10^−10.97^	0.72
T = 100 °C	6.1 × 10^−5^	0	0	T = 100 °C	1.8 × 10^−6^	10^−10.58^	0.69
T = 160 °C	5.3 × 10^−5^	0	0	T = 160 °C	4.5 × 10^−6^	10^−9.89^	0.62

## Data Availability

The data presented in this study are available on request from the corresponding author.

## Supplementary Materials

The following are available online at https://www.mdpi.com/article/10.3390/nano11051093/s1, Supplementary Figure S1: Spin-coating process (feeding of dissolved metal solution onto ITO substrate using micropipette); Supplementary Figure S2: FE-SEM images for (a) CuIn_0.4_Ga_0.6_Se_2_, (b) CuIn_0.4_Cr_0.1_Ga_0.5_Se_2_, (c) CuIn_0.4_Cr_0.2_Ga_0.4_Se_2_, and (d) CuIn_0.4_Cr_0.3_Ga_0.3_Se^2^ nano-crystalline precursor powders at 2 μm; Supplementary Figure S3: EDX charts for (a) CuIn_0.4_Ga_0.6_Se_2_, (b) CuIn_0.4_Cr_0.1_Ga_0.5_Se_2_, (c) CuIn_0.4_Cr_0.2_Ga_0.4_Se_2_, and (d) CuIn_0.4_Cr_0.3_Ga_0.3_Se_2_ precursor powders.
