# Supplementary material for: Structural and Electrochemical Analysis of CIGS: Cr Crystalline Nanopowders and Thin Films Deposited onto ITO Substrates
Source: Nanomaterials (Basel). 2021 Apr 23;11(5):1093. doi: 10.3390/nano11051093 (PMC8146074; doi:10.3390/nano11051093)
Supplement: Supplementary file 1 [file nanomaterials-11-01093-s001.zip › nanomaterials-1167393-supplementary.pdf]

nanomaterials

Supporting Information for

Article

Structural and Electrochemical Analysis of CIGS: Cr Crystalline Nanopowders and Thin Films Deposited onto ITO Substrates

Suzan Saber [1,2], Bernabé Marí [1,*], Andreu Andrio [3], Jorge Escorihuela [4,*], Nagwa Khattab [2], Ali Eid [2], Amany El Nahrawy [2], Mohamed Abo Aly [5], and Vicente Compañ [6,*]

[1] Institut de Disseny i Fabricació, Universitat Politècnica de València, Camí de Vera s/n, 46022 Valencia, Spain; s.k.saber@hotmail.com
[2] National Research Center, 33 El Bohouth St. (former El Tahrir St.), Dokki, Giza, Cairo 12622, Egypt; nag_khb@yahoo.com (N.K.); suzankamal85@gmail.com (A.E.); amany_physics_1980@yahoo.com (A.E.N.)
[3] Departament de Física Aplicada, Universitat Jaume I; Avda. Sos Baynat s/n, 12080 Castelló, Spain; andrio@uji.es
[4] Departamento de Química Orgánica, Universitat de València; Avda. Vicent Andrés Estellés s/n, Burjassot, 46100 Valencia, Spain
[5] Chemistry Department, Faculty of Science, Ain Shams University; Cairo 11566, Egypt; aboalymoh@hotmail.com
[6] Departament de Termodinàmica Aplicada, Escola Tècnica Superior d'Enginyers Industrials (ETSII), Universitat Politècnica de València, Camí de Vera s/n, 46022 València, Spain
* Correspondence: bmari@fis.upv.es (B.M.); jorge.escorihuela@uv.es (J.E.); vicommo@ter.upv.es (V.C.)

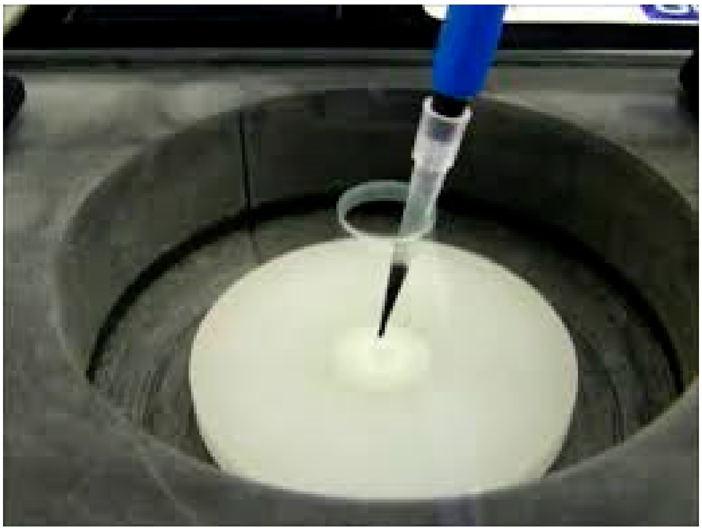

Figure 1. Spin-coating process (feeding of dissolved metal solution onto ITO substrate using micropipette).

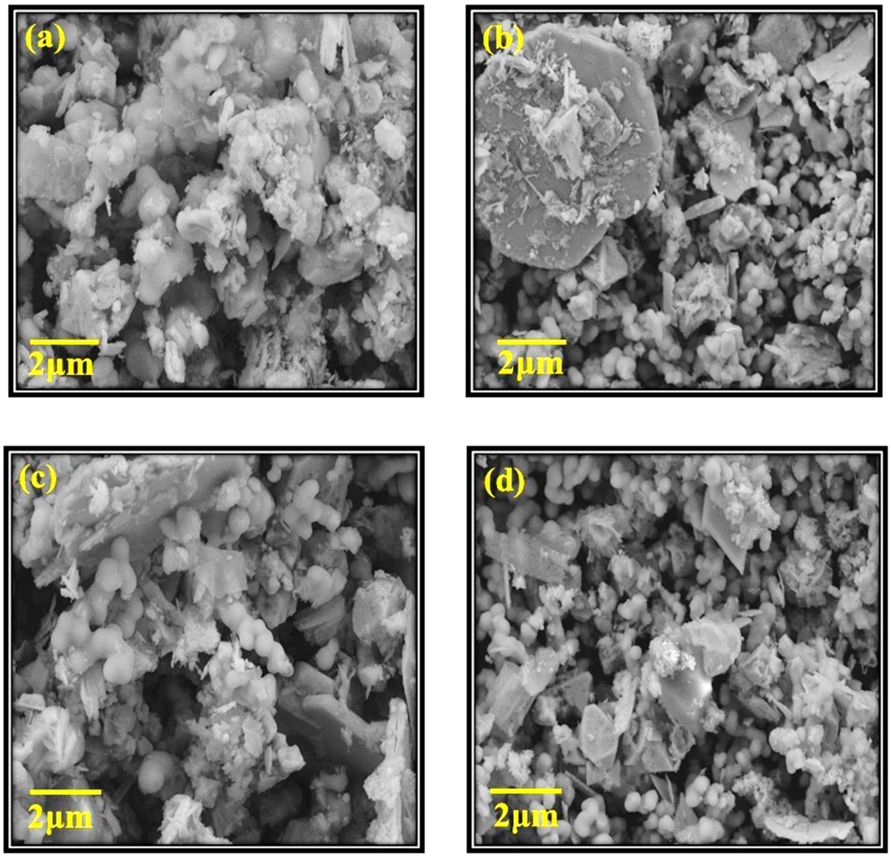

Figure S2. FE-SEM images for (**a**) $CuIn_{0.4}Ga_{0.6}Se_2$, (**b**) $CuIn_{0.4}Cr_{0.1}Ga_{0.5}Se_2$, (**c**) $CuIn_{0.4}Cr_{0.2}Ga_{0.4}Se_2$, and (**d**) $CuIn_{0.4}Cr_{0.3}Ga_{0.3}Se_2$ nanocrystalline precursor powders at 2µm.

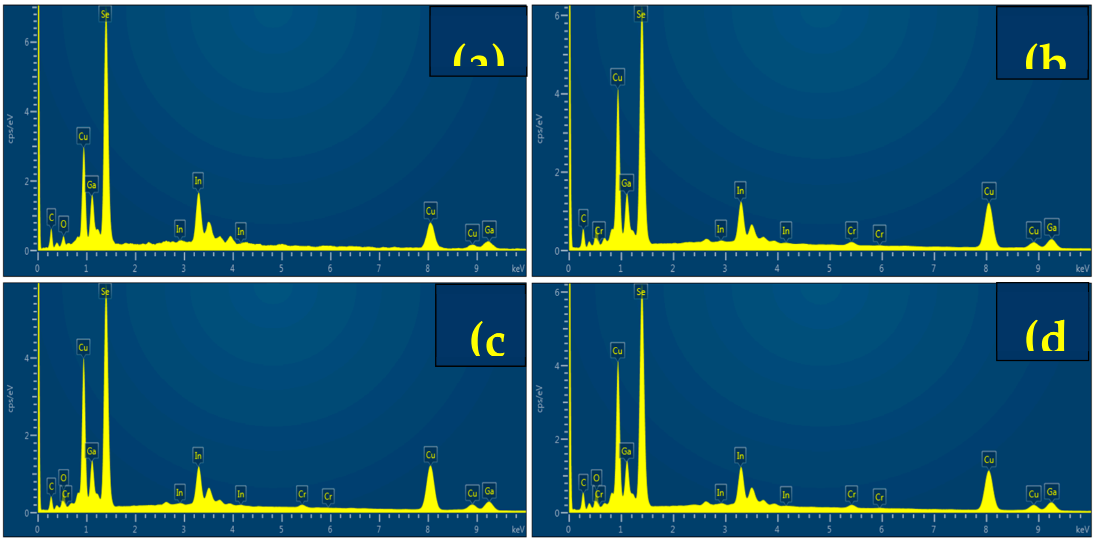

Figure 3. EDX charts for (**a**) $CuIn_{0.4}Ga_{0.6}Se_2$, (**b**) $CuIn_{0.4}Cr_{0.1}Ga_{0.5}Se_2$, (**c**) $CuIn_{0.4}Cr_{0.2}Ga_{0.4}Se_2$, and (**d**) $CuIn_{0.4}Cr_{0.3}Ga_{0.3}Se_2$ precursor powders.
